# Tolerogenic Dendritic Cells on Transplantation: Immunotherapy Based on Second Signal Blockage

**DOI:** 10.1155/2015/856707

**Published:** 2015-10-12

**Authors:** Priscila de Matos Silva, Julia Bier, Lisiery Negrini Paiatto, Cassia Galdino Albuquerque, Caique Lopes Souza, Luis Gustavo Romani Fernandes, Wirla Maria da Silva Cunha Tamashiro, Patricia Ucelli Simioni

**Affiliations:** ^1^Department of Biomedical Science, Faculty of Americana (FAM), 13477-360 Americana, SP, Brazil; ^2^Medical School, University of Campinas (UNICAMP), 13083-887 Campinas, SP, Brazil; ^3^Department of Genetics, Evolution and Bioagents, Institute of Biology, University of Campinas (UNICAMP), 13083-970 Campinas, SP, Brazil; ^4^Department of Biochemistry and Microbiology, Institute of Biosciences, Universidade Estadual Paulista (UNESP), 13506-900 Rio Claro, SP, Brazil

## Abstract

Dendritic cells (DCs), the most important professional antigen-presenting cells (APC), play crucial role in both immunity and tolerance. It is well known that DCs are able to mount immune responses against foreign antigens and simultaneously tolerate self-antigens. Since DCs can be modulated depending on the surrounding microenvironment, they can act as a bridge between innate and adaptive immunity. However, the mechanisms that support this dual role are not entirely clear. Recent studies have shown that DCs can be manipulated *ex vivo* in order to trigger their tolerogenic profile, what can be a tool to be used in clinical trials aiming the treatment of various diseases and the prevention of transplant rejection. In this sense, the blockage of costimulatory molecules on DC, in the attempt of inhibiting the second signal in the immunological synapse, can be considered as one of the main strategies under development. This review brings an update on current therapies using tolerogenic dendritic cells modulated with costimulatory blockers with the aim of reducing transplant rejection. However, although there are current clinical trials using tolerogenic DC to treat allograft rejection, the actual challenge is to modulate these cells in order to maintain a permanent tolerogenic profile.

## 1. Background

The main goal of a successful transplant is to promote immune tolerance of the transplanted organ or tissue, allowing the reestablishment of normal physiological functions, without generating damage to the recipient or to the transplanted tissue. The concept of tolerance in transplantation is understood as a state in which no pathological immune response is generated against the transplanted organ or tissue. This condition would make the graft viable while retaining the necessary immune responses against other unknown antigens [1, 2]. Thereby, the relationship between tolerance and immunity must be well balanced, since any alteration in one of the parts can cause pathophysiological modifications and, consequently, can trigger changes in the immune system that can ultimately lead to autoimmunity or graft rejection [3]. In this context, it is known that a successful transplant relies on a deep understanding of the immune system allied with the balance and maintenance of effector and regulatory immune mechanisms [1, 4].

However, even successful transplants can have severe long-term complications, which can culminate in allograft rejection. Several immunossupressor treatments have been developed in order to reduce transplant rejection. However, despite significant advances on immunosuppressive strategies, antirejection drugs still present serious side effects, such as high susceptibility of opportunistic infectious diseases, or even inefficient suppression of immune responses against the allograft. The knowledge acquisition about the immune regulation mechanisms, especially about the role of the antigen-presenting cells (APC) in tolerance, can help researchers propose new strategies and immunotherapies to prevent rejection [5].

**Figure 1:**
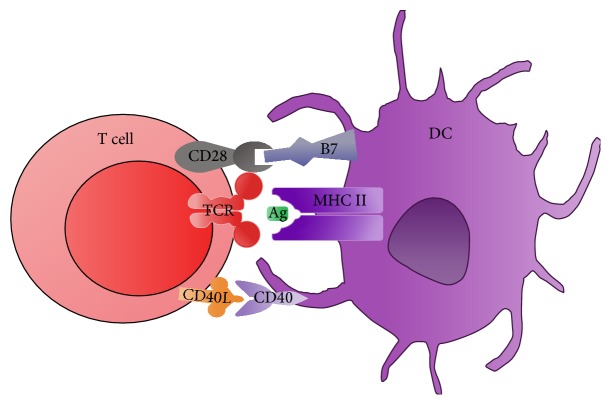
Schematic representation of the DC and T cell interaction: the main costimulatory molecules. Activation of T cell involves both interactions between the T cell costimulatory receptors, CD28 with their cognate ligands, CD80, and CD86 (B7 family) as well as the CD40L/CD40 pathway. Other costimulatory molecules, such as OX40/OX40-L and TIM-1 and PD-1/PD-L1, were not represented here. DC: dendritic cell; MHC II: major histocompatibility complex II; TCR: T cell receptor; CD40L: CD40 ligand.

Among the APC, dendritic cells (DCs) represent the first line of immune cell defense against pathogens and constitute a bridge between innate and adaptive immune response. As represented in Figure 1, DCs are the most important APC for naive T cells [5–8] and can exert either immunogenic or tolerogenic functions. Depending on the received signals, these cells can become tolerogenic, that is, can inhibit antigen-specific immune response [7, 9–13]. When TCR interacts with the peptide-MHC (pMHC) on the surface of the APC (first signal) and it is not followed by the interaction between costimulatory molecules (second signal), it can induce anergy on T cells [14]. Dendritic cells express important costimulators to T cell activation, such as the B7 family molecules: CD80 (B7-1) and CD86 (B7-2), playing an important role in either tolerogenic or immunogenic responses. Therefore, the handling of costimulatory molecules, aiming the application of DC for therapeutic purposes in immune disorders such as allergies and autoimmunities, as well as in vaccination and transplantation, has received extensive attention [15].

In this sense, in the attempt of modulating the activity of DC on the treatment of autoimmunity, hypersensibility, and transplant rejection, many researchers aim to develop therapies based on tolerogenic DC (tol-DC). Previous data has shown that DC modulated by interleukin- (IL-) 10 or transforming growth factor-beta (TGF-*β*) became refractory to sustain the* in vitro *proliferation of antigen-specific effector T lymphocytes [12, 13]. Additionally, adoptive transfer of DC, modulated by inhibitory cytokines such as IL-10, also leads to a reduction of* in vivo *delayed-type hypersensibility (DTH) responses [16].

Apart from DC, regulatory T (Treg) cells, particularly CD4^+^ CD25^+^ Foxp3^+^ lymphocytes, play an important role in inducing and maintaining tolerance, promoted by cell to cell contact or by secreted cytokines such as IL-10 and TGF-*β* [17–19].

In this review, we focus our attention on current knowledge related to immunotherapeutic advances based on the use of tolerogenic DC through inhibition of the second signal, which contribute to increasing survival of transplanted organs and tissues and reducing the use of immunosuppressive drugs.

## 2. Innate Immune System on Graft Rejection

Even though the role of the adaptive immune system through cellular and humoral responses in transplant rejection is well known, many researchers have outlined the involvement of components of the innate immune system in the mechanisms of alloreactivity and rejection. Among these components, the most studied are the toll-like receptors (TLR), complement system, natural killer (NK) cells, DC, granulocytes, and inflammatory cytokines which perform different functions in innate immune responses [20–22].

In this regard, TLR links innate and adaptive immunity and its signaling leads to the transcription of genes involved in inflammatory responses resulting in the production of proinflammatory cytokines and chemokines, antimicrobial peptides, adhesion molecules, enhanced antigen presentation, and upregulation of costimulatory molecules in APC [20, 21]. Corroborating this fact, it was demonstrated in mouse models of graft versus host disease (GVHD) that elevated doses of radiation during pretransplant increase the epithelial damage of mucosal tissues, allowing bacterial components to pass through the barrier. These components activate host and/or donor APC by interacting with pattern recognition receptors (PRR), such as TLR, converting these APC into an activated profile that is able to prime alloreactive donor-derived T cells, resulting in a more severe GVHD [23].

Currently, complement activation is known to occur in transplant rejection and contribute to progression of rejection. Specifically, activation of the lectin pathway of the complement system is implicated in allograft rejection. Also, the role of complement system in modulating regulatory T cells is under investigation [24–27]. Recent study suggests that C3a and C5a signaling promotes cell proliferation of activated T cells and reduces induced-Treg (iTreg) generation and stability [28].

Macrophages were considered critical components in both acute cellular allograft rejection and chronic injury [29]. However, it is already known that macrophages can play both detrimental and beneficial functions in allograft rejection. In initial stages of the transplant, the immune response can create a proinflammatory microenvironment that favors the differentiation of M1 macrophages (previously referred to as classically or alternatively activated macrophages). M1 phenotype is a proinflammatory cell type, characterized by secretion of proinflammatory cytokines, high phagocytic activity, and production of reactive oxygen species. As inflammation recedes, this may alter the milieu to favor M2 differentiation. M2 macrophages have an immunomodulatory role, since they produce IL-10, presenting reduced phagocytic activity and increased arginase production [30].

This hypothesis was supported by an assay with CCR5^−/−^ mice that exhibited reduced macrophage accumulation after transplantation. In this model, M2 macrophage activation was increased while M1 macrophage activation was reduced after transplantation, in comparison with control mice [31]. However, a recent study with human biopsies showed controversial data, since a M2 macrophage infiltration was associated with inflammation, injury, and fibrosis in renal allograft [32, 33].

Additionally, NK cells are an important component of the innate immune system, helping to recognize allogeneic MHC and are capable of producing proinflammatory cytokines. NK cells can impair tolerance induction in a solid organ transplant and can also lead to acute and chronic rejection of allogeneic transplant [34–36]. It happens because NK cells can kill either directly donor cells through granzyme, perforin, Fas ligand (FasL), and tumor necrosis factor-related apoptosis-inducing ligand (TRAIL) pathways [37–40] or indirectly by lysing Treg cells or by promoting CD4^+^ T cell activation [36, 41, 42].

Controversially, NK and NKT cells have been known to be enrolled on allograft tolerance induction. This role of NK cells can be influenced by immunosuppressive therapies such as calcineurin inhibitors or steroids [35, 43, 44]. The suppressive role of NK cells has been observed on the allorecognition suppression of T cells by a perforin-dependent mechanism [45] and also by killing the donor antigen-presenting cells [46]. Also, NK cells can regulate macrophage activation in transplanted tissue or organ by a mechanism partially dependent on an activating receptor known as natural killer group 2, member D (NKG2D) [47].

Invariant NKT (iNKT) cells have regulatory functions on the Th1/Th2 imbalance by releasing Th2 cytokines such as IL-4 and IL-5 that antagonize the Th1 responses related with acute rejection [48, 49]. Also, repeated activation of NKT cells by *α*-galactosylceramide leads to IL-10 production [50, 51]. Therefore, the success of tolerance protocols in transplantation will require the administration of agents capable of suppressing innate immunity as well as adaptive immunity [52].

### 2.1. Tolerogenic Potential of Dendritic Cells on Graft Rejection

Dendritic cells are a potential tool for therapeutic applications in transplantation and strategies that promote DC tolerogenicity are under evaluation [53–56]. The discovery of DC's function is considered a landmark in the field of immunology, since it plays a relevant role in the interaction between innate and adaptive immune response. In 1973, Steinman and Cohn characterized and named DCs, a key population in naive T lymphocyte activation [57]. These studies originated from the necessity of a better understanding of how antigens could activate T cells and how this activation contributed to the effector mechanisms of the immune response. Steinman's studies have demonstrated that DC can be functionally characterized by the presence of high levels of MHC expression. Soon after, the importance of the DCs' maturation stage for their immunogenic or tolerogenic functions became clear [58]. These APC, widely distributed in lymphoid tissues, mucosal epithelium, and parenchymal organs [58, 59], are originating from myeloid or lymphoid precursors in the bone marrow and circulate in the bloodstream as immature cells before migrating to peripheral tissues [59]. In innate immunity, these cells respond to pathogen-associated molecular patterns (PAMPs) of microbes by generating and secreting inflammatory cytokines. In adaptive immunity, these cells process and present antigen to T cells that leads to their activation [60].

In humans, two major subpopulations of DC were characterized: conventional DC (cDC) and plasmacytoid DC (pDC). When stimulated by microbial antigens via TLR-2 and TLR-4, cDC produces large amounts of IL-12 that drives immune response to a Th1 profile. Conventional DC also activates cytotoxic lymphocytes (CTL) in a process known as cross-presentation. On the other hand, pDC has a lower capacity of antigen uptake. However, pDC expresses intracellular TLR 7 and TLR 9, which detect ssRNA and CpG DNA motifs, respectively. pDC's main function is to initiate antiviral responses producing large amounts of type I interferons such as IFN-*α* and INF-*β*. Together, cDC and pDC can distinctively contribute to protecting the host against pathogens [61, 62].

In an inflammatory microenvironment, DCs initiate their maturation process by undergoing changes in their morphology that facilitate the interaction with naive T cells. The hallmark of the mature stage is the upregulation of MHC and costimulatory molecules on DC surface. Another relevant factor is that DCs dramatically increase their migratory capacity due to the augmentation in chemokines expression, a process that occurs by the upregulation of the CCR7 receptor and their interaction with two major chemokines, CCL19 and CCL21. Naive T cells also express CCR7 and, as DC, migrate to the lymph node regions, thus increasing the likelihood of interacting between APC and naive T lymphocyte [63]. The matured and activated DCs cease to recognize and process antigens, consequently preventing the presentation of self-antigens at the site of inflammation. In summary, the set of events that occur during maturation can mold DCs as highly effective inducers of proliferation and differentiation of naive T cells [63].

After exposure to antigen and crosstalk with T cells, DCs express high levels of costimulatory molecules and cytokines. In this regard, tolerogenic- (tol-) DC can be generated by altering these signals [64–66]. In order to achieve a tolerogenic profile, a DC must be immature, which means that the maturation degree of a DC can determine its tolerogenic capacity. According to this, the immature DC expresses low levels of MHC class II and low levels of costimulatory molecules, such as CD40, CD80, and CD86, and, consequently, it presents a low capacity of activating T cells, which is potentially associated with T cell anergy and increased Tregs generation. In this context, immature DC has demonstrated its ability of negatively regulating immunogenic responses to alloantigen in animal models [67, 68].

It is well known that immature cDC generated* in vitro* from bone marrow cells in the presence of the granulocyte-macrophage-colony stimulating factor (GM-CSF) administered seven days before the heterotopic cardiac transplantation graft in rats produces a significant increase in survival time of the graft. This primarily occurs because the immature DC presents a reduced expression of costimulatory molecules [69].

Regarding the secretion of cytokines, immature tol-DC can also be generated* in vitro* in the presence of specific cytokines. One of the major cytokines that contribute to the generation of tol-DC is the IL-10. DC modulated by IL-10 inhibits the expression of MHC class II and of CD80 and CD86 costimulatory molecules, acquiring the ability to induce T cell anergy. IL-2, secreted mainly by activated T CD4^+^ lymphocytes, also plays an important role in maintaining tolerance by regulatory T cells, since these cells are highly dependent on IL-2 for their functions [59].

The literature indicates that the tolerogenic DC populations have specific markers related to their tolerogenic capacity as well as high expression and activity of indoleamine 2,3-dioxygenase (IDO), a tryptophan-catabolizing enzyme [70]. The suppressive mechanism related to IDO is associated with massive depletion of tryptophan, serotonin, and melatonin in the tissue microenvironment, producing immunoregulatory metabolites, the kynurenines [71]. Besides, other metabolites derived from tryptophan by IDO activity can also foster the generation of Treg cells, demonstrating the important immunosuppressive role of IDO, either by direct suppression of T cell activation, or expansion of Treg cells. Data shows that IDO has a greater potential to protect the tissue from damage than to prevent the activation of T cells [70, 72].

## 3. Adaptive Immunity on Graft Rejection

### 3.1. Cellular Basis of Allograft Rejection

Much of what is currently known about allograft rejection is mainly related to the understanding of the effective role of T cells in alloantigen recognition mechanisms. However, innate-adaptive immune crosstalk is fundamental in this process. The participation of T cells in the recognition of alloantigen occurs through the interaction between the receptors of T lymphocytes and allogeneic MHC expressed on APC [73], promoting the differentiation of naive T cells into effector T cells [74]. The migration of naive T cells to lymphoid organs is mediated by specific chemokines, such as intercellular adhesion molecules (ICAM) as well as chemokine (C-C motif) ligand 21 (CCL21), which allow the migration of lymphocytes through blood vessels. In lymphoid organs, naive T lymphocytes can encounter DC bearing antigen molecules. Accordingly, the naive T cells initiate their differentiation depending on the signal intensity that can then become effector cells [75].

The CD4^+^ helper T cells promote the activation of macrophages by the production of specific cytokines, also assisting in the differentiation of plasmocytes and consequently in the production of antibodies. The helper T cells promote the expansion of memory CD8^+^ T cells after secondary exposure to antigen [76]. Many factors, including cytokines, influence the differentiation of naive CD4^+^ T cells. The major subsets of T helper effector cells are Th1, Th2, Th9, Th17, Th22, and follicular helper T cells [77, 78]. Among them, Th1 cells are one of the most important populations responsible for immune response evolved with the allograft rejection, while the role of Th2 cells is controversial; some data support their involvement in the activation of alloimmune responses while other data shows their contribution as a regulatory subset [79].

More recently, the Th17 population stands out as an important cell group related with inflammatory conditions. In this sense, the influence of Th17 cells in the activation of inflammatory conditions in GVHD in patients was demonstrated. These cells migrate to GVHD target organs, as skin and mucosa, and exert their proinflammatory effects, stimulating the Th1 effector cells migration to these sites [80].

It has been shown that another heterogeneous subset of Foxp3^+^Treg cells promotes the inhibition of the activation of T lymphocytes, balancing the intensity of immune responses [75, 81]. In allogenic cardiac mouse model, the adoptive transfer of Foxp3^+^Treg cells induced* in vitro* by exposition of naïve T lymphocytes to tolerogenic DC was able to provide long-term tolerance and allograft survival [82]. In agreement with these findings, Hu and collaborators [83] showed that infiltrating Foxp3^+^Treg cells found in kidney allografts from mouse seem to be related with the tolerance phenomenon whereas the depletion of these cells correlates with tolerance abrogation and decreased graft survival. However, it has been demonstrated that Foxp3^+^Treg and Th17 cells populations have a high flexibility and lineage plasticity, being able to convert from one to another by a mechanism dependent on the retinoic acid receptor-related orphan receptor *γ* (ROR*γ*t) [84, 85]. Supporting this, a recent investigation in a mouse cardiac transplantation model showed that the transference of mesenchymal stem cells (MSC) before heart transplantation was able to induce Treg over Th17 development. According to the authors, the identification of IL-17A+ Foxp3+ double-positive and ex-IL-17-producing IL-17A-Foxp3+ T cells in heart and spleen of the recipients argues for direct conversion of Th17 cells into Treg cells as the underlying mechanism of immune regulation in MSC-mediated allograft survival [85].

Among immunological molecules related to allograft response, human leukocyte antigen (HLA)-G, a nonclassical class of I HLA detected in the plasma, has been associated with the reduction of acute and chronic rejection [86]. This molecule has local immunomodulatory properties, due to its limited polymorphism, contributing to the survival of allogeneic liver transplants. HLA-G1 to G4 are membrane-bound molecules while HLA-G5 to G7 are soluble molecules (sHLA-G) [87, 88]. HLA-G was previously identified as a naturally occurring tolerance-inducing molecule. Under physiological condition, HLA-G has a low tissue distribution, being mainly found in medullary thymic epithelial cells, cornea, pancreas, and mesenchymal stem cells. Their tolerogenic functions were observed during pregnancy for preventing maternal NK cytotoxicity and suppressing the activation and proliferation of CD4 and CD8 T cells [89, 90]. It is already known that low doses of sHLA can stimulate Th2 and inhibit Th1 profile [91, 92]. However, it was also demonstrated that sHLA induced proliferation and IFN-*γ* production by NK cells, contributing to vascular remodeling of spiral arteries and allowing successful embryo implantation in pregnancy model [92].

### 3.2. Humoral Basis of Allograft Rejection

Antibody-mediated immune response, described in the literature as hyperacute graft rejection, occurs mainly in highly vascularized organs transplanted into previously sensitized recipients. This phenomenon can occur in distinct conditions: when patients might have received multiple blood transfusions, when they have been pregnant, or when they could possibly have had a previous transplant treatment. All these situations would explain why they would be carrying antibodies against donor antigens (DSAs). The result is a hyperacute rejection mediated by specific antibodies due to an incompatibility between donor and recipient, manifested by a strong reaction against the donor HLA antigens in the vascular endothelial cells of the graft [93].

The interaction between graft endothelial cells and host antibodies provides rapid complement activation and subsequent graft loss. This is caused by a serious inflammatory injury in the endothelium, losing its capacity of retaining fluid within the intravascular space [94–96]. Hereupon, the evolution of therapies for reducing the impact of B cells and DSA is a goal on allograft survival.

## 4. Immunotherapies Targeting Allograft Rejection

The establishment of an effective standard therapy for the induction of tolerance in the prevention of graft rejection is of great complexity. Hence, the long-term graft survival is often dependent on the maintenance of immunosuppressive treatment, which generates serious side effects [97, 98]. Rather, it would be interesting to have treatments with milder side effects such as tolerance induction therapy [4, 99], highlighting the importance of creating protocols based on nonaggressive immunosuppressive drugs.

In this sense, the progression of research and immunological knowledge allowed the development of new immunosuppressive drugs and molecules based on animal model studies. The literature describes a wide range of tolerogenic therapeutics, many of which are still under experimental studies. However, some biological therapies have shown considerable success in allogeneic transplant, at least in the short term [100–102]. Thereby, therapies based on inducing cellular tolerance have become important alternatives for reducing the administration of immunosuppressive drugs in the attempt of improving the life quality of transplanted patients [4, 99]. However, more studies are necessary to investigate the risks associated with modern cellular therapies, since they can be related to an increased number of malignancy and infectious diseases [103, 104].

### 4.1. Immunotherapy Based on the Second Signal Blockage

As previously mentioned, the secondary signaling has a great importance to the T cell immune response and can be a relevant tool to the development of immunological tolerance. Thereby, new immunotherapies are under evaluation for the treatment of autoimmune diseases, hypersensibility, and transplantation. Several studies have focused on the generation of tol-DC by blocking the costimulatory pathways, as summarized in Table 1 [5, 98, 105].

Among costimulatory molecules, T cells express the CD28 receptor on their surface as the main responsible molecule for binding to the B7-1 and B7-2 (CD80 and CD86, resp.) receptors, present on the surfaces of APC. The interaction between these molecules promotes the differentiation and activation of T effector cells, together with the production of associated cytokines, triggering the immune response [106, 107]. Naive T cells highly express CD28 molecule which avidly interacts with B7 molecules present on APC. The interaction between CD28L/B7 induces the secretion of IL-2 and interferes with the tolerogenic property of immature DC. This occurs primarily by decreasing the induction of regulatory T cells and also by leveraging the differentiation of effector T cells [63].

On the other hand, the cytotoxic T Lymphocyte Antigen-4 (CTLA4) molecule, also known as CD152, acts as an inhibitory receptor of the immune response; that is, it blocks the binding site between CD28 and B7, providing a negative signal to T lymphocytes, thus inhibiting the immune response [148, 149]. Also, the inhibitory signals released by the interaction between CTLA4 and B7 result in an increased secretion of immunomodulatory cytokines, such as IL-10 and TGF-*β*, and hence the generation of Treg cells [108, 150, 151]. Suppression or anergy, induced by CTLA4, are associated with Treg functions [152]. A recent research has shown that CTLA4 molecule can also be found on DC. The cross-linking of CTLA4 can inhibit the maturation of DC, playing an inhibitory role in immune response [153].

Since CD80/CD86 molecules present a higher affinity for CTLA4 rather than for CD28 molecule on the T cells, the binding of CTLA4 and B7 family molecules makes it possible to achieve tolerance to the allograft [152, 154]. It has been demonstrated that CTLA4-deficient mice exhibit severe autoimmune phenotype with early death 3 to 4 weeks after birth, resulting from the massive destruction of multiple organs, demonstrating the fundamental role of CTLA4 in the regulation of peripheral self-reactive T cells [155].

**Figure 2:**
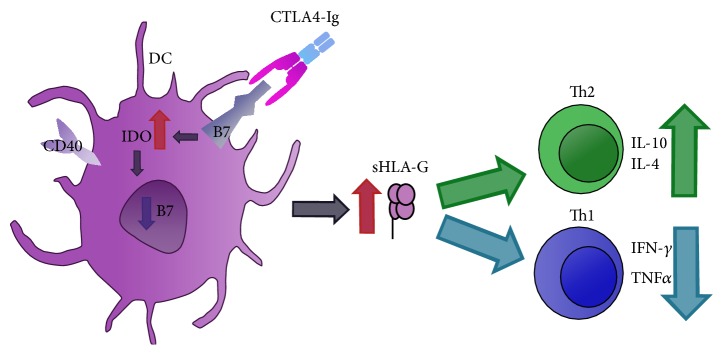
Mechanism of action of CTLA4-Ig on DC. CTLA4-Ig soluble molecule binds to B7 (CD80/CD86) molecules on DC. CTLA4 presents a higher affinity to B7 molecule and competes with CD28 for this ligation. This interaction induces downregulation of B7 gene transcription and upregulation of IDO as well as secretion of sHLA-G. sHLA-G can stimulate Th2 and inhibit Th1 profile. DC: dendritic cell; CTLA4-Ig: extracellular domain of human CTLA4 with a portion of the Fc region of IgG; IDO: indoleamine 2,3-dioxygenase; sHLA-G: soluble HLA-G; Th: T helper; IL: interleukin; IFN: interferon; TNF: tumor necrosis factor.

Based on this knowledge and on the attempt of modulating the immune response in allografts, recombinant molecules of CTLA4 linked to immunoglobulins (Ig) have been developed (CTLA4-Ig). These molecules, termed fusion protein antagonist CTLA4-Ig, combine the extracellular domain of human CTLA4 with a portion of the Fc region of IgG. As represented in Figure 2, the CTLA4-Ig, initially tested for the treatment of rheumatoid arthritis, has shown greater affinity for B7, acting directly on APC and optimizing the inhibition of the immune response [107, 108]. The inhibition mechanism can also be related to the fact that CTLA4-Ig-treated DC suppresses T cell proliferation through sHLA-G secretion. Additionally, CTLA4-Ig induces IDO expression in DC [88, 92, 156]. sHLA-G was associated with an increase in the number of regulatory T cells and a shift of cytokine towards Th2 [157].

In this context, Abatacept, a commercial CTLA4-Ig, selectively modulates the immune response by binding with high affinity to the B7 family present on APC. Thus, this drug inhibits the activation of T cells by competing by the binding site of CD28 receptors, preventing the secondary signal from occurring [109, 110]. In human model, the T cell hyporesponsiveness was also associated with a higher expression of a negative regulator of proliferation, named p27kip1 (cyclin-dependent kinase inhibitor 1B [CDKN1B]) [111]. Another recombinant molecule with an altered form, Belatacept, has been approved for its use in transplants. This molecule is known for having major affinity against CD86 [112–114]. A study with kidney-transplanted patients treated with Belatacept demonstrates that transplanted patients who received treatment with CTLA4-Ig had higher plasma levels of sHLA-G. It can be hypostasized that the immunosuppressive action of sHLA-G isoforms in transplants is associated with the suppression of allogeneic T cells expansion and the inhibition of the activation of both CD8^+^ T cells and NK cells [88, 154]. In summary, the inhibitory signals carried by the blockage of the CTLA4 molecule with CTLA4-Ig can be related to the attenuation of stimulatory signal, decreasing the cell proliferation, and cell cycle progression and alteration in cytokine production of effector T cells.

**Figure 3:**
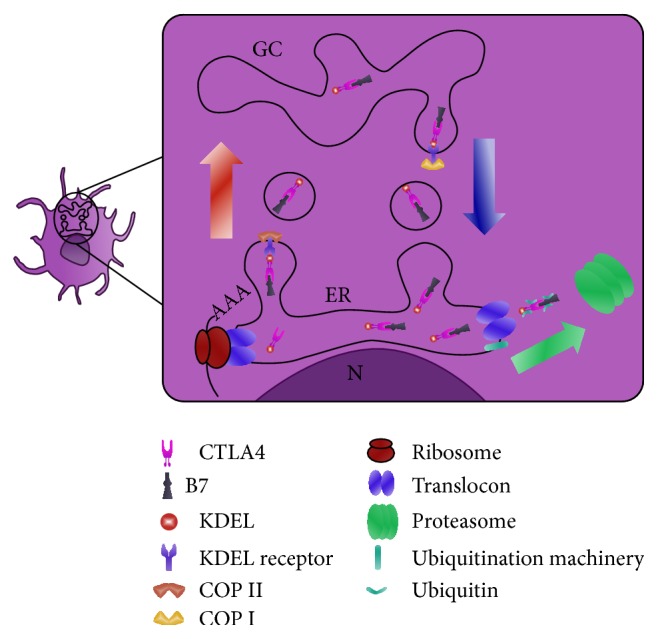
Hypothetical mechanism of action of CTLA4-KDEL fusion protein. Transport of proteins between the ER and Golgi apparatus is mediated by two membrane coat complexes, COPI and COPII. COPII mediates ER-to-Golgi transport and COPI mediates retrograde transport. KDEL receptor undergoes retrograde transport only after it binds its ligand [147]. On CTLA4-KDEL transfected cells, the KDEL peptide retains/retrieves proteins to the ER. CTLA4 fused to KDEL is confined to the ER where it binds CD80/86, preventing the passage of these molecules to the cell surface. CD80/CD86 molecules seem to be removed by proteasome-mediated degradation. GC: complex of Golgi; N: nucleus; ER: endoplasmatic reticulum; CTLA4-KDEL: gene construct encoding a modified CTLA4 molecule; COP: cytosolic protein coat complex.

As schematized in Figure 3, CTLA4 fused to the endoplasmic reticulum retention/retrieval signal sequence named KDEL (CTLA4-KDEL) is a fusion protein that targets the endoplasmic reticulum (ER). CTLA4-KDEL is confined to ER and binds to CD80/86, preventing their passage to the cell surface by interaction with receptors. APC expressing this construct retain CD80/CD86 molecules in the endoplasmic reticulum and fail to express these costimulatory molecules on their surface [158–160]. A recent study demonstrated the applicability of modulating the signal transduction in murine DC with CTLA4-KDEL in order to inhibit immune response in corneal transplantation. CTLA4-KDEL-expressing DC adopted a tolerogenic phenotype and induced anergy in alloreactive T cells, both* in vitro* and* in vivo*, resulting in a long-term survival of corneal allografts [72]. CTLA4-Ig in DC cultures showed the expected reduction in IFN-*γ* and IL-4 which may be associated with the upregulation of IDO in DCs, not seen in CTLA4-KDEL-transfected cells [72, 115].

**Figure 4:**
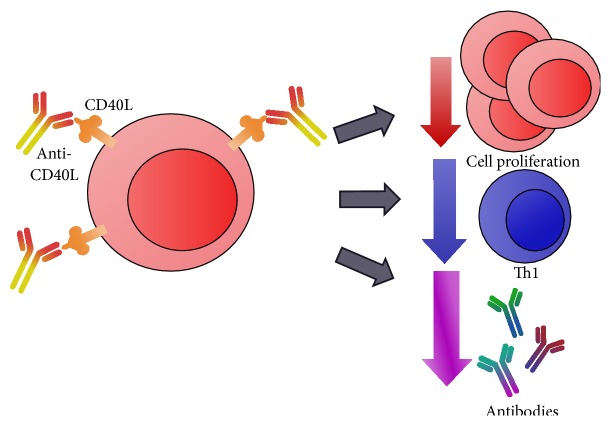
Anti-CD40L interaction and effects on T cells. Costimulatory molecule CD40L is primarily expressed on activated CD4^+^ T lymphocytes. Anti-CD40L binds to the CD40L present on T cell and blocks its interaction with CD40 receptor present on APC. Therapy with anti-CD40L (CD154) or anti-CD40 MAb, alone or combined with other molecules, downregulates T cell proliferation, Th1 cytokine production, and antibody secretion. CD40L: CD40 ligand; APC: antigen-presenting cell.

The interactions between CD40 and CD40L, expressed on APC and T cells, respectively, are strictly related with allograft immune response. As represented in Figure 4, it has been shown that the blockage of the CD40-CD40L interaction improves allograft survival by preventing the occurrence of acute rejection [116, 124, 125, 161]. Therapy with anti-CD40L (CD154) MAb prolonged the survival of the corneal allograft by increasing the frequency of spleen IL-10^+^CD4^+^ T cells and decreasing IFN-*γ* ^+^CD4^+^ T cells. Also, the Treg/Th1 cell ratio was increased in experimental model [162]. Since therapies with anti-CD40 or anti-CD40L MAb presented several thromboembolic complications in clinical applications, further studies are ongoing to evaluate the combination of these molecules [126, 130, 161]. Accordingly, the therapy of CTLA4-Ig and a nondepleting CD40 monoclonal antibody, named 3A8, is a promising combination [126], since preliminary data showed an increased duration of graft acceptance with this immunosuppressive treatment [126, 127].

Programmed death- (PD-) 1 ligand (PD-L1 or B7-H1) and PD-L2 (B7-DC) are new B7 family members expressed on APC. PD-1 and PD-1L/PD-L2 costimulatory signals play important roles in T cell induced immune responses; PD-L1 and PD-L2 deliver inhibitory signals that regulate T cell activation and tolerance [136]. In a corneal allograft model, PD-1 prolonged transplant survival by PD-L1 interaction [137]. In this sense, dimeric PD-L1 immunoglobulin (Ig) fusion protein (PD-L1.Ig) seems to be another combinatory therapy in transplants as in corneal allograft, where PD-L1.Ig showed significant suppression on T cell activation [137, 138, 163]. However, further studies will be required to determine the therapeutic property of this molecule.

Additionally, T cell immunoglobulin domain and mucin domain (TIM) family is a newly discovered group of molecules that regulate immune cell function. TIM-1 molecule is expressed on T cells and APC [139]. The interaction between TIM-1 and TIM-4 promotes Th2 responses, and the blockage of this interaction can decrease allergic responses [140, 141]. RMT1-10, an anti-TIM-1 monoclonal antibody, was effective in blocking TIM-1 and in promoting corneal allograft survival in mice [141].

There are other coadjutant costimulatory blocking molecules that are under evaluation. Anti-OX40-L MAb therapy prevents memory T cell-mediated cardiac allograft vasculopathy in mice, suggesting a potential therapy for inhibition of OX40-OX40L signaling [128, 129, 142]. This MAb seems to act by reducing the pool of effector T cells responses, most part of these being CD8^+^ T cells [143].

Human leukocyte function antigen-3 (LFA-3) is an adhesion and costimulation molecule, found on a variety of APC, which interacts with ligand CD2 on T cells. LFA3-Ig (Alefacept), a humanized chimeric fusion protein, comprises an extracellular CD2-binding portion of the LFA3 linked to the Fc portion of a human IgG1. LFA3-Ig promotes renal allograft survival by depleting CD8^+^ effector memory T cells and interfering with T cell activation [113, 144, 145]. Also, this molecule can activate Fc gamma R(+) cells, such as NK cells, to induce apoptosis of sensitive CD2(+) target cells [144, 146].

### 4.2. Other Modulatory Agents on Tolerogenic Dendritic Cell

A new approach in the attempt of combining treatments with autologous tolerogenic DC and anti-CD3 antibodies is under development. This therapy was shown to be effective in mice with pancreatic islet allografts by providing a reduction of T cells infiltration. However, these protocols are still under clinical development [164, 165].

Alternative methods for modulating tol-DC using tolerance-inducing agents such as dexamethasone (Dexa), rapamycin (Rapa), and vitamin D3 (VitD3) are also under evaluation [54]. Dexa-treated-DC triggered by lipopolysaccharide (LPS) led to the suppression of proliferative response of primed T cells, triggering the differentiation of various populations of Tregs [166]. In another experimental study, BALB/c mice that received a corneal transplantation were treated with an analogue of resolvin D1 (RvD1). Resolvin D1 is a lipid mediator that plays an important role in resolution of acute inflammation. RvD1 modulated DC showed a significant reduction in maturation. Also, interferon-gamma-secreting T cell frequency was decreased and alloimmune sensitization was reduced after transplantation [167].

## 5. Concluding Remarks

Organ and tissue transplantation is still a last resource, being only considered in cases such as total organ failure. Thus, avoidance of rejection of transplanted organs is a key task. Current allograft therapies can cause many side effects; hence several alternative therapies, aiming the induction of tolerance, mainly based on infusion of DC and Tregs, have been proposed in the attempt of aiding this scenario. Accordingly, immature DC expressing low levels of MHC and costimulatory molecules has been considered among the treatments due to its low capacity to activate T cells, thus promoting a natural immunosuppression, which reduces the need of using immunosuppressive drugs.

The modulation of DC with CTLA4-Ig has shown positive effects on suppressing the immune response. Although many studies involving fusion proteins, and even monoclonal antibodies, are in early stages, this is a very promising tool and has great clinical potential in reducing transplant rejection. Among the promising treatments, the effectiveness of using CTLA4-Ig in immune modulation and in the induction of tol-DC has been shown, even though the use of tolerogenic cells for therapeutic purposes on transplantation is still not widely available in clinical practice.

Essentially, the main challenge in these therapies is to fixate the DC phenotype, since tol-DC can only be determinate by its tolerogenic effect. Thus, the fine control of the subtle balance between immunization and tolerance by DC is necessary to allow the use of DC in clinical practice.

Despite being a very promising therapy, studies of adverse effects should be extensive, since the use of biotechnology in medical treatment, in the transplantation scenario in particular, can be very risky if not thoroughly understood.

## Figures and Tables

**Table 1:** Immunotherapy based on the second signal blockage.

Molecule	Commercial name/clone	Target molecule	Mechanism of action	References
CTLA4-Ig	Abatacept/Belatacept	CD80/CD86	Competition for binding to B7 molecules on DC Upregulation of IDO enzyme expression High plasma levels of sHLA-G High secretion of immunomodulatory cytokines Inhibition of CD8^+^ T cells and NK cells Generation of Treg cells Inhibition of proliferation related to p27kip1 expression	[88, 107–123]

CTLA4-KDEL	—	CD80/CD86	Retention of costimulatory molecules within the ER T cell anergy by an IDO-independent way	[72, 115]

Anti-CD40L (CD154)/anti-CD40	3A8, 4D11, ASKP1240, 7E1	CD40/CD40L	Inhibition of antibody secretion Downregulation of T cell proliferation Inhibition of cytokine secretion and costimulatory molecule synthesis Upregulation of spleen IL-10^+^CD4^+^; T cells and downregulation of IFN-*γ* ^+^CD4^+^ T cells	[117, 123–135]

PD-L1.Ig	—	PD-1/PD-1L	Suppression of T cell activation	[136–138]

Anti-TIM-1	RMT1-10	TIM-1	Blockage of TIM-1 ligation	[139–141]

Anti-OX40-L	—	OX40-OX40L interaction	Inhibition of OX40-OX40L signaling Prevention of T memory cells Reduction of effector T cells	[128, 129, 142, 143]

LFA3-Ig	Alefacept	LFA3 (CD58)	Depletion of CD8^+^ effector memory T cells Reduction of T cell activation	[113, 116, 144–146]

## References

[1] Ashoor I. F., Najafian N. (2012). Rejection and regulation: a tight balance. *Current Opinion in Organ Transplantation*.

[2] Lee K., Nguyen V., Lee K.-M., Kang S.-M., Tang Q. (2014). Attenuation of donor-reactive T cells allows effective control of allograft rejection using regulatory T cell therapy. *American Journal of Transplantation*.

[3] Pan P.-Y., Ozao J., Zhou Z., Chen S.-H. (2008). Advancements in immune tolerance. *Advanced Drug Delivery Reviews*.

[4] McDonald-Hyman C., Turka L. A., Blazar B. R. (2015). Advances and challenges in immunotherapy for solid organ and hematopoietic stem cell transplantation. *Science Translational Medicine*.

[5] Moreau A., Varey E., Bouchet-Delbos L., Cuturi M.-C. (2012). Cell therapy using tolerogenic dendritic cells in transplantation. *Transplantation Research*.

[6] Steinman R. M., Gutchinov B., Witmer M. D., Nussenzweig M. C. (1983). Dendritic cells are the principal stimulators of the primary mixed leukocyte reaction in mice. *The Journal of Experimental Medicine*.

[7] Banchereau J., Steinman R. M. (1998). Dendritic cells and the control of immunity. *Nature*.

[8] Steinman R. M., Hawiger D., Nussenzweig M. C. (2003). Tolerogenic dendritic cells. *Annual Review of Immunology*.

[9] Finkelman F. D., Lees A., Birnbaum R., Gause W. C., Morris S. C. (1996). Dendritic cells can present antigen in vivo in a tolerogenic or immunogenic fashion. *Journal of Immunology*.

[10] Nagler-Anderson C., Terhoust C., Bhan A. K., Podolsky D. K. (2001). Mucosal antigen presentation and the control of tolerance and immunity. *Trends in Immunology*.

[11] Legge K. L., Gregg R. K., Maldonado-Lopez R. (2002). On the role of dendritic cells in peripheral T cell tolerance and modulation of autoimmunity. *Journal of Experimental Medicine*.

[12] Belz G. T., Heath W. R., Carbone F. R. (2002). The role of dendritic cell subsets in selection between tolerance and immunity. *Immunology and Cell Biology*.

[13] Kapsenberg M. L. (2003). Dendritic-cell control of pathogen-driven T-cell polarization. *Nature Reviews Immunology*.

[14] Mueller D. L., Jenkins M. K., Schwartz R. H. (1989). Clonal expansion versus functional clonal inactivation: a costimulatory signalling pathway determines the outcome of T cell antigen receptor occupancy. *Annual Review of Immunology*.

[15] Ardavín C. (2003). Origin, precursors and differentiation of mouse dendritic cells. *Nature Reviews Immunology*.

[16] Müller G., Müller A., Tüting T. (2002). Interleukin-10-treated dendritic cells modulate immune responses of naive and sensitized T cells In vivo. *Journal of Investigative Dermatology*.

[17] Nakamura K., Kitani A., Strober W. (2001). Cell contact-dependent immunosuppression by CD4^+^CD25^+^ regulatory T cells is mediated by cell surface-bound transforming growth factor *β*. *The Journal of Experimental Medicine*.

[18] Thorstenson K. M., Khoruts A. (2001). Generation of anergic and potentially immunoregulatory CD25^+^CD4 T cells in vivo after induction of peripheral tolerance with intravenous or oral antigen. *The Journal of Immunology*.

[19] Ramsdell F. (2003). Foxp3 and natural regulatory T cells: key to a cell lineage?. *Immunity*.

[20] Benichou G., Tonsho M., Tocco G., Nadazdin O., Madsen J. C. (2012). Innate immunity and resistance to tolerogenesis in allotransplantation. *Frontiers in Immunology*.

[21] LaRosa D. F., Rahman A. H., Turka L. A. (2007). The innate immune system in allograft rejection and tolerance. *Journal of Immunology*.

[22] Li X. C. (2010). The significance of non-T-cell pathways in graft rejection: implications for transplant tolerance. *Transplantation*.

[23] Heidegger S., van den Brink M. R. M., Haas T., Poeck H. (2014). The role of pattern-recognition receptors in graft-versus-host disease and graft-versus-leukemia after allogeneic stem cell transplantation. *Frontiers in Immunology*.

[24] Farrar C. A., Sacks S. H. (2014). Mechanisms of rejection: role of complement. *Current Opinion in Organ Transplantation*.

[25] Regele H., Böhmig G. A., Habicht A. (2002). Capillary deposition of complement split product C4d in renal allografts is associated with basement membrane injury in peritubular and glomerular capillaries: a contribution of humoral immunity to chronic allograft rejection. *Journal of the American Society of Nephrology*.

[26] Smedbråten Y. V., Sagedal S., Mjøen G. (2015). High ficolin-3 level at the time of transplantation is an independent risk factor for graft loss in kidney transplant recipients. *Transplantation*.

[27] Murata K., Baldwin W. M. (2009). Mechanisms of complement activation, C4d deposition, and their contribution to the pathogenesis of antibody-mediated rejection. *Transplantation Reviews*.

[28] Cravedi P., van der Touw W., Heeger P. S. (2013). Complement regulation of T-cell alloimmunity. *Seminars in Nephrology*.

[29] Mannon R. B. (2012). Macrophages: contributors to allograft dysfunction, repair, or innocent bystanders?. *Current Opinion in Organ Transplantation*.

[30] Kwan T., Wu H., Chadban S. J. (2014). Macrophages in renal transplantation: roles and therapeutic implications. *Cellular Immunology*.

[31] Dehmel S., Wang S., Schmidt C. (2010). Chemokine receptor Ccr5 deficiency induces alternative macrophage activation and improves long-term renal allograft outcome. *European Journal of Immunology*.

[32] Toki D., Zhang W., Hor K. L. M. (2014). The role of macrophages in the development of human renal allograft fibrosis in the first year after transplantation. *American Journal of Transplantation*.

[33] Ikezumi Y., Suzuki T., Yamada T. (2015). Alternatively activated macrophages in the pathogenesis of chronic kidney allograft injury. *Pediatric Nephrology*.

[34] Oertel M., Kohlhaw K., Diepolder H. M. (2001). Alloreactivity of natural killer cells in allogeneic liver transplantation. *Transplantation*.

[35] Pratschke J., Stauch D., Kotsch K. (2009). Role of NK and NKT cells in solid organ transplantation. *Transplant International*.

[36] Kitchens W. H., Uehara S., Chase C. M., Colvin R. B., Russell P. S., Madsen J. C. (2006). The changing role of natural killer cells in solid organ rejection and tolerance. *Transplantation*.

[37] Trapani J. A., Smyth M. J. (2002). Functional significance of the perforin/granzyme cell death pathway. *Nature Reviews Immunology*.

[38] Smyth M. J., Cretney E., Takeda K. (2001). umor necrosis factor-related apoptosis-inducing ligand (TRAIL) contributes to interferon *γ*-dependent natural killer cell protection from tumor metastasis. *The Journal of Experimental Medicine*.

[39] Zhang Y., Cheng G., Xu Z.-W. (2013). Down regulation of TRAIL and FasL on NK cells by Cyclosporin A in renal transplantation patients. *Immunology Letters*.

[40] Zhang Z.-X., Huang X., Jiang J. (2014). Natural killer cells play a critical role in cardiac allograft vasculopathy in an interleukin-6—dependent manner. *Transplantation*.

[41] Ito A., Shimura H., Nitahara A. (2008). NK cells contribute to the skin graft rejection promoted by CD4^+^ T cells activated through the indirect allorecognition pathway. *International Immunology*.

[42] Uehara S., Chase C. M., Kitchens W. H. (2005). NK cells can trigger allograft vasculopathy: the role of hybrid resistance in solid organ allografts. *The Journal of Immunology*.

[43] Meehan A. C., Mifsud N. A., Nguyen T. H. O. (2013). Impact of commonly used transplant immunosuppressive drugs on human NK cell function is dependent upon stimulation condition. *PLoS ONE*.

[44] Leyking S., Budich K., van Bentum K. (2015). Calcineurin inhibitors differentially alter the circadian rhythm of T-cell functionality in transplant recipients. *Journal of Translational Medicine*.

[45] Beilke J. N., Kuhl N. R., Van Kaer L., Gill R. G. (2005). NK cells promote islet allograft tolerance via a perforin-dependent mechanism. *Nature Medicine*.

[46] Yu G., Xu X., Vu M. D., Kilpatrick E. D., Li X. C. (2006). NK cells promote transplant tolerance by killing donor antigen-presenting cells. *The Journal of Experimental Medicine*.

[47] Van Der Touw W., Burrell B., Lal G., Bromberg J. S. (2012). NK cells are required for costimulatory blockade induced tolerance to vascularized allografts. *Transplantation*.

[48] Tsuruyama T., Fujimoto Y., Yonekawa Y. (2012). Invariant natural killer T cells infiltrate intestinal allografts undergoing acute cellular rejection. *Transplant International*.

[49] Liu Y., Luan X., Li J., He Y., Li M. (2012). The role of invariant NKT cells in liver transplant tolerance in rats. *Transplantation Proceedings*.

[50] Jiang X., Kojo S., Harada M., Ohkohchi N., Taniguchi M., Seino K.-I. (2007). Mechanism of NKT cell-mediated transplant tolerance. *American Journal of Transplantation*.

[51] Seino K.-I., Fukao K., Muramoto K. (2001). Requirement for natural killer T (NKT) cells in the induction of allograft tolerance. *Proceedings of the National Academy of Sciences of the United States of America*.

[52] Brennan T. V., Rendell V. R., Yang Y. (2015). Innate immune activation by tissue injury and cell death in the setting of hematopoietic stem cell transplantation. *Frontiers in Immunology*.

[53] Morelli A. E., Thomson A. W. (2014). Orchestration of transplantation tolerance by regulatory dendritic cell therapy or in-situ targeting of dendritic cells. *Current Opinion in Organ Transplantation*.

[54] Naranjo-Gómez M., Raïch-Regué D., Oñate C. (2011). Comparative study of clinical grade human tolerogenic dendritic cells. *Journal of Translational Medicine*.

[55] Raïch-Regué D., Glancy M., Thomson A. W. (2014). Regulatory dendritic cell therapy: from rodents to clinical application. *Immunology Letters*.

[56] Gordon J. R., Ma Y., Churchman L., Gordon S. A., Dawicki W. (2014). Regulatory dendritic cells for immunotherapy in immunologic diseases. *Frontiers in Immunology*.

[57] Steinman R. M., Cohn Z. A. (1973). Identification of a novel cell type in peripheral lymphoid organs of mice. I. Morphology, quantitation, tissue distribution. *The Journal of Experimental Medicine*.

[58] Katsnelson A. (2006). Kicking off adaptive immunity: the discovery of dendritic cells. *Journal of Experimental Medicine*.

[59] Amodio G., Gregori S. (2012). Human tolerogenic DC-10: perspectives for clinical applications. *Transplantation Research*.

[60] Van Brussel I., Berneman Z. N., Cools N. (2012). Optimizing dendritic cell-based immunotherapy: tackling the complexity of different arms of the immune system. *Mediators of Inflammation*.

[61] Ezzelarab M., Thomson A. W. (2011). Tolerogenic dendritic cells and their role in transplantation. *Seminars in Immunology*.

[62] van Montfoort N., van der Aa E., Woltman A. M. (2014). Understanding MHC class I presentation of viral antigens by human dendritic cells as a basis for rational design of therapeutic vaccines. *Frontiers in Immunology*.

[63] Hubo M., Trinschek B., Kryczanowsky F., Tuettenberg A., Steinbrink K., Jonuleit H. (2013). Costimulatory molecules on immunogenic versus tolerogenic human dendritic cells. *Frontiers in Immunology*.

[64] Kalantari T., Kamali-Sarvestani E., Ciric B. (2011). Generation of immunogenic and tolerogenic clinical-grade dendritic cells. *Immunologic Research*.

[65] Zobywalski A., Javorovic M., Frankenberger B. (2007). Generation of clinical grade dendritic cells with capacity to produce biologically active IL-12p70. *Journal of Translational Medicine*.

[66] Tan S. M., Kapp M., Flechsig C. (2013). Stimulating surface molecules, Th1-polarizing cytokines, proven trafficking—a new protocol for the generation of clinical-grade dendritic cells. *Cytotherapy*.

[67] Turnquist H. R., Raimondi G., Zahorchak A. F., Fischer R. T., Wang Z., Thomson A. W. (2007). Rapamycin-conditioned dendritic cells are poor stimulators of allogeneic CD4^+^ T cells, but enrich for antigen-specific Foxp3^+^ T regulatory cells and promote organ transplant tolerance. *The Journal of Immunology*.

[68] Manicassamy S., Pulendran B. (2011). Dendritic cell control of tolerogenic responses. *Immunological Reviews*.

[69] Fu F., Li Y., Qian S. (1996). Costimulatory molecule-deficient dendritic cell progenitors (MHC class II+, CD80dim, CD86-) prolong cardiac allograft survival in nonimmunosuppressed recipients. *Transplantation*.

[70] Harden J. L., Egilmez N. K. (2012). Indoleamine 2,3-dioxygenase and dendritic cell tolerogenicity. *Immunological Investigations*.

[71] Fallarino F., Grohmann U., You S. (2006). The combined effects of tryptophan starvation and tryptophan catabolites down-regulate T cell receptor zeta-chain and induce a regulatory phenotype in naive T cells. *Journal of Immunology*.

[72] Khan A., Fu H., Tan L. A. (2013). Dendritic cell modification as a route to inhibiting corneal graft rejection by the indirect pathway of allorecognition. *European Journal of Immunology*.

[73] Smith K. A. (2012). Toward a molecular understanding of adaptive immunity: a chronology part II. *Frontiers in Immunology*.

[74] Meuer S. C., Schlossman S. F., Reinherz E. L. (1982). Clonal analysis of human cytotoxic T lymphocytes: T4^+^ and T8^+^ effector T cells recognize products of different major histocompatibility complex regions. *Proceedings of the National Academy of Sciences of the United States of America*.

[75] Magombedze G., Reddy P. B. J., Eda S., Ganusov V. V. (2013). Cellular and population plasticity of helper CD4^+^ T cell responses. *Frontiers in Physiology*.

[76] Prlic M., Williams M. A., Bevan M. J. (2007). Requirements for CD8 T-cell priming, memory generation and maintenance. *Current Opinion in Immunology*.

[77] Bluestone J. A., Mackay C. R., O'Shea J. J., Stockinger B. (2009). The functional plasticity of T cell subsets. *Nature Reviews Immunology*.

[78] Gerlach K., Hwang Y., Nikolaev A. (2014). TH9 cells that express the transcription factor PU.1 drive T cell-mediated colitis via IL-9 receptor signaling in intestinal epithelial cells. *Nature Immunology*.

[79] Abdoli R., Najafian N. (2014). T helper cells fate mapping by co-stimulatory molecules and its functions in allograft rejection and tolerance. *International Journal of Organ Transplantation Medicine*.

[80] Van der Waart A. B., van der Velden W. J. F. M., Blijlevens N. M., Dolstra H. (2014). Targeting the IL17 pathway for the prevention of graft-versus-host disease. *Biology of Blood and Marrow Transplantation*.

[81] Liston A., Gray D. H. D. (2014). Homeostatic control of regulatory T cell diversity. *Nature Reviews Immunology*.

[82] Takasato F., Morita R., Schichita T. (2014). Prevention of allogeneic cardiac graft rejection by transfer of ex vivo expanded antigen-specific regulatory T-cells. *PLoS ONE*.

[83] Hu M., Wang C., Zhang G. Y. (2013). Infiltrating Foxp3^+^ regulatory T cells from spontaneously tolerant kidney allografts demonstrate donor-specific tolerance. *American Journal of Transplantation*.

[84] Zhou L., Lopes J. E., Chong M. M. W. (2008). TGF-beta-induced Foxp3 inhibits T(H)17 cell differentiation by antagonizing RORgammat function. *Nature*.

[85] Obermajer N., Popp F. C., Soeder Y. (2014). Conversion of Th17 into IL-17A^neg^ regulatory T cells: a novel mechanism in prolonged allograft survival promoted by mesenchymal stem cell-supported minimized immunosuppressive therapy. *The Journal of Immunology*.

[86] Hu W.-Y., Wu L.-Q., Su Z., Pang X.-F., Zhang B. (2014). Expression of human leukocyte antigen-G and acute rejection in patients following liver transplantation. *Experimental and Therapeutic Medicine*.

[87] Azarpira N., Aghdaie M. H., Kazemi K., Geramizadeh B., Darai M. (2014). HLA-G polymorphism (rs16375) and acute rejection in liver transplant recipients. *Disease Markers*.

[88] Bahri R., Naji A., Menier C. (2009). Dendritic cells secrete the immunosuppressive HLA-G molecule upon CTLA4-Ig treatment: implication in human renal transplant acceptance. *The Journal of Immunology*.

[89] Rouas-Freiss N., Gonçalves R. M.-B., Menier C., Dausset J., Carosella E. D. (1997). Direct evidence to support the role of HLA-G in protecting the fetus from maternal uterine natural killer cytolysis. *Proceedings of the National Academy of Sciences of the United States of America*.

[90] Colonna M., Samaridis J., Cella M. (1998). Human myelomonocytic cells express an inhibitory receptor for classical and nonclassical MHC class I molecules. *Journal of Immunology*.

[91] Kapasi K., Albert S. E., Yie S.-M., Zavazava N., Librach C. L. (2000). HLA-G has a concentration-dependent effect on the generation of an allo-CTL response. *Immunology*.

[92] van der Meer A., Lukassen H. G. M., van Cranenbroek B. (2007). Soluble HLA-G promotes Th1-type cytokine production by cytokine-activated uterine and peripheral natural killer cells. *Molecular Human Reproduction*.

[93] Krishnan N. S., Zehnder D., Briggs D., Higgins R. (2012). Human leukocyte antigen antibody incompatible renal transplantation. *Indian Journal of Nephrology*.

[94] Colvin R. B., Smith R. N. (2005). Antibody-mediated organ-allograft rejection. *Nature Reviews Immunology*.

[95] Wasowska B. A. (2010). Mechanisms involved in antibody- and complement-mediated allograft rejection. *Immunologic Research*.

[96] Duijvestijn A. M., Derhaag J. G., van Breda Vriesman P. J. C. (2000). Complement activation by anti-endothelial cell antibodies in MHC-mismatched and MHC-matched heart allograft rejection: anti-MHC-, but not anti non-MHC alloantibodies are effective in complement activation. *Transplant International*.

[97] Meier-Kriesche H.-U., Schold J. D., Kaplan B. (2004). Long-term renal allograft survival: have we made significant progress or is it time to rethink our analytic and therapeutic strategies?. *American Journal of Transplantation*.

[98] Cobbold S. P., Li X. C. (2012). Translating tolerogenic therapies to the clinic—where do we stand and what are the barriers?. *Frontiers in Immunology*.

[99] Salama A. D., Womer K. L., Sayegh M. H. (2007). Clinical transplantation tolerance: many rivers to cross. *Journal of Immunology*.

[100] Sayegh M. H., Carpenter C. B. (2004). Transplantation 50 years later—progress, challenges, and promises. *The New England Journal of Medicine*.

[101] Linden P. K. (2009). History of solid organ transplantation and organ donation. *Critical Care Clinics*.

[102] De Serres S. A., Sayegh M. H., Najafian N. (2009). Immunosuppressive drugs and tregs: a critical evaluation!. *Clinical Journal of the American Society of Nephrology*.

[103] Ferrer I. R., Hester J., Bushell A., Wood K. J. (2014). Induction of transplantation tolerance through regulatory cells: from mice to men. *Immunological Reviews*.

[104] Tey S.-K. (2014). Adoptive T-cell therapy: adverse events and safety switches. *Clinical & Translational Immunology*.

[105] Volchenkov R., Karlsen M., Jonsson R., Appel S. (2013). Type 1 regulatory T cells and regulatory B cells induced by tolerogenic dendritic cells. *Scandinavian Journal of Immunology*.

[106] Tuettenberg A., Huter E., Hubo M. (2009). The role of ICOS in directing T cell responses: ICOS-dependent induction of T cell anergy by tolerogenic dendritic cells. *Journal of Immunology*.

[107] Herrero-Beaumont G., Calatrava M. J. M., Castañeda S. (2012). Abatacept mechanism of action: concordance with its clinical profile. *Reumatología Clínica*.

[108] Ruderman E. M., Pope R. M. (2005). The evolving clinical profile of abatacept (CTLA4-Ig): a novel co-stimulatory modulator for the treatment of rheumatoid arthritis. *Arthritis Research and Therapy*.

[109] Korhonen R., Moilanen E. (2009). Abatacept, a novel CD80/86-CD28 T cell co-stimulation modulator, in the treatment of rheumatoid arthritis. *Basic and Clinical Pharmacology and Toxicology*.

[110] Koura D. T., Horan J. T., Langston A. A. (2013). In vivo T cell costimulation blockade with abatacept for acute graft-versus-host disease prevention: a first-in-disease trial. *Biology of Blood and Marrow Transplantation*.

[111] Rochman Y., Yukawa M., Kartashov A. V., Barski A. (2015). Functional characterization of human T cell hyporesponsiveness induced by CTLA4-Ig. *PLoS ONE*.

[112] Shen J., Townsend R., You X. (2014). Pharmacokinetics, pharmacodynamics, and immunogenicity of belatacept in adult kidney transplant recipients. *Clinical Drug Investigation*.

[113] Lowe M. C., Badell I. R., Turner A. P. (2013). Belatacept and sirolimus prolong nonhuman primate islet allograft survival: adverse consequences of concomitant alefacept therapy. *American Journal of Transplantation*.

[114] Larsen C. P., Grinyó J., Medina-Pestana J. (2010). Belatacept-based regimens versus a cyclosporine a-based regimen in kidney transplant recipients: 2-year results from the benefit and benefit-EXT studies. *Transplantation*.

[115] Tan P. H., Yates J. B., Xue S.-A. (2005). Creation of tolerogenic human dendritic cells via intracellular CTLA4: a novel strategy with potential in clinical immunosuppression. *Blood*.

[116] Thompson P., Badell I. R., Lowe M. (2012). Alternative immunomodulatory strategies for xenotransplantation: CD40/154 pathway-sparing regimens promote xenograft survival. *American Journal of Transplantation*.

[117] Gilson C. R., Milas Z., Gangappa S. (2009). Anti-CD40 monoclonal antibody synergizes with CTLA4-Ig in promoting long-term graft survival in murine models of transplantation. *Journal of Immunology*.

[118] Cutolo M., Nadler S. G. (2013). Advances in CTLA-4-Ig-mediated modulation of inflammatory cell and immune response activation in rheumatoid arthritis. *Autoimmunity Reviews*.

[119] Ko H.-J., Cho M.-L., Lee S.-Y. (2010). CTLA4-Ig modifies dendritic cells from mice with collagen-induced arthritis to increase the CD4^+^CD25^+^Foxp3^+^ regulatory T cell population. *Journal of Autoimmunity*.

[120] Mayer E., Hölzl M., Ahmadi S. (2013). CTLA4-Ig immunosuppressive activity at the level of dendritic cell/T cell crosstalk. *International Immunopharmacology*.

[121] Lan Y. Y., Wang Z., Raimondi G. (2006). ‘Alternatively activated’ dendritic cells preferentially secrete IL-10, expand Foxp3^+^CD4^+^ T cells, and induce long-term organ allograft survival in combination with CTLA4-Ig. *The Journal of Immunology*.

[122] Lan Y. Y., Wang Z., Raimondi G. Organ Allograft Survival in Combination with CTLA4-Ig 1.

[123] Emmanouilidis N., Guo Z., Dong Y. (2006). Immunosuppressive and trafficking properties of donor splenic and bone marrow dendritic cells. *Transplantation*.

[124] Lina L. U., Wei L. I., Fumin F. U. (1997). Blockade of the CD40-CD40 ligand pathway potentiates the capacity of donor-derived dendritic cell progenitors to induce long-term cardiac allograft survival. *Transplantation*.

[125] Nathan M. J., Mold J. E., Wood S. C. (2004). Requirement for donor and recipient CD40 expression in cardiac allograft rejection: induction of Th1 responses and influence of donor-derived dendritic cells. *The Journal of Immunology*.

[126] Page A., Srinivasan S., Singh K. (2012). CD40 blockade combines with CTLA4Ig and sirolimus to produce mixed chimerism in an MHC-defined rhesus macaque transplant model. *American Journal of Transplantation*.

[127] Badell I. R., Russell M. C., Cardona K. (2012). CTLA4Ig prevents alloantibody formation following nonhuman primate islet transplantation using the CD40-specific antibody 3A8. *American Journal of Transplantation*.

[128] Dai H., Peng F., Lin M. (2015). Anti-OX40L monoclonal antibody prolongs secondary heart allograft survival based on CD40/CD40L and LFA-1/ICAM-1 blockade. *Transplant Immunology*.

[129] Wang H., Zhang Z., Tian W. (2014). Memory T cells mediate cardiac allograft vasculopathy and are inactivated by Anti-OX40L monoclonal antibody. *Cardiovascular Drugs and Therapy*.

[130] Aoyagi T., Yamashita K., Suzuki T. (2009). A human anti-CD40 monoclonal antibody, 4D11, for kidney transplantation in cynomolgus monkeys: induction and maintenance therapy. *The American Journal of Transplantation*.

[131] Watanabe M., Yamashita K., Suzuki T. (2013). ASKP1240, a fully human anti-CD40 monoclonal antibody, prolongs pancreatic islet allograft survival in nonhuman primates. *The American Journal of Transplantation*.

[132] Sun W., Wang Q., Zhang L. (2003). Blockade of CD40 pathway enhances the induction of immune tolerance by immature dendritic cells genetically modified to express cytotoxic T lymphocyte antigen 4 immunoglobulin. *Transplantation*.

[133] Nathan M. J., Yin D., Eichwald E. J., Bishop D. K. (2002). The immunobiology of inductive anti-CD40L therapy in transplantation: allograft acceptance is not dependent upon the deletion of graft-reactive T cells. *American Journal of Transplantation*.

[134] Larsen C. P., Alexander D. Z., Hollenbaugh D. (1996). CD40-gp39 interactions play a critical role during allograft rejection: suppression of allograft rejection by blockade of the CD40-gp39 pathway. *Transplantation*.

[135] Shimizu K., Schönbeck U., Mach F., Libby P., Mitchell R. N. (2000). Host CD40 ligand deficiency induces long-term allograft survival and donor-specific tolerance in mouse cardiac transplantation but does not prevent graft arteriosclerosis. *The Journal of Immunology*.

[136] Keir M. E., Butte M. J., Freeman G. J., Sharpe A. H. (2008). PD-1 and its ligands in tolerance and immunity. *Annual Review of Immunology*.

[137] Watson M. P., George A. J. T., Larkin D. F. P. (2006). Differential effects of costimulatory pathway modulation on corneal allograft survival. *Investigative Ophthalmology and Visual Science*.

[138] Li T., Ma R., Zhu J. Y., Wang F. S., Huang L., Leng X. S. (2015). PD-1/PD-L1 costimulatory pathway-induced mouse islet transplantation immune tolerance. *Transplantation Proceedings*.

[139] Rennert P. D. (2011). Novel roles for TIM-1 in immunity and infection. *Immunology Letters*.

[140] Li Z., Ju Z., Frieri M. (2013). The T-cell immunoglobulin and mucin domain (Tim) gene family in asthma, allergy, and autoimmunity. *Allergy and Asthma Proceedings*.

[141] Tan X., Jie Y., Zhang Y., Qin Y., Xu Q., Pan Z. (2014). Tim-1 blockade with RMT1-10 increases T regulatory cells and prolongs the survival of high-risk corneal allografts in mice. *Experimental Eye Research*.

[142] Turgeon N. A., Avila J. G., Cano J. A. (2010). Experience with a novel efalizumab-based immunosuppressive regimen to facilitate single donor islet cell transplantation. *The American Journal of Transplantation*.

[143] Kinnear G., Wood K. J., Marshall D., Jones N. D. (2010). Anti-OX40 prevents effector T-cell accumulation and CD8+ T-cell mediated skin allograft rejection. *Transplantation*.

[144] Lee S., Yamada Y., Tonsho M. (2013). Alefacept promotes immunosuppression-free renal allograft survival in nonhuman primates via depletion of recipient memory T cells. *The American Journal of Transplantation*.

[145] Rostaing L., Charpentier B., Glyda M. (2013). Alefacept combined with tacrolimus, mycophenolate mofetil and steroids in de novo kidney transplantation: a randomized controlled trial. *American Journal of Transplantation*.

[146] da Silva A. J., Brickelmaier M., Majeau G. R. (2002). Alefacept, an immunomodulatory recombinant LFA-3/IgG1 fusion protein, induces CD16 signaling and CD2/CD16-dependent apoptosis of CD2^+^ cells. *Journal of Immunology*.

[147] Orci L., Stamnes M., Ravazzola M. (1997). Bidirectional transport by distinct populations of COPI-coated vesicles. *Cell*.

[148] Wang X., Hao J., Metzger D. L. (2011). B7-H4 pathway in islet transplantation and *β*-cell replacement therapies. *Journal of Transplantation*.

[149] Corse E., Allison J. P. (2012). Cutting edge: CTLA-4 on effector T cells inhibits in trans. *Journal of Immunology*.

[150] Perez N., Karumuthil-Melethil S., Li R., Prabhakar B. S., Holterman M. J., Vasu C. (2008). Preferential costimulation by CD80 results in IL-10-dependent TGF-beta1(+) -adaptive regulatory T cell generation. *The Journal of Immunology*.

[151] Pletinckx K., Döhler A., Pavlovic V., Lutz M. B. (2011). Role of dendritic cell maturity/costimulation for generation, homeostasis, and suppressive activity of regulatory T cells. *Frontiers in Immunology*.

[152] Tai X., Van Laethem F., Pobezinsky L. (2012). Basis of CTLA-4 function in regulatory and conventional CD4^+^ T cells. *Blood*.

[153] Wang X. B., Fan Z. Z., Anton D. (2011). CTLA4 is expressed on mature dendritic cells derived from human monocytes and influences their maturation and antigen presentation. *BMC Immunology*.

[154] Wu Y. L., Liang J., Zhang W., Tanaka Y., Sugiyama H. (2012). Immunotherapies: the blockade of inhibitory signals. *International Journal of Biological Sciences*.

[155] Jain N., Nguyen H., Chambers C., Kang J. (2010). Dual function of CTLA-4 in regulatory T cells and conventional T cells to prevent multiorgan autoimmunity. *Proceedings of the National Academy of Sciences of the United States of America*.

[156] López A. S., Alegre E., LeMaoult J., Carosella E., González Á. (2006). Regulatory role of tryptophan degradation pathway in HLA-G expression by human monocyte-derived dendritic cells. *Molecular Immunology*.

[157] Rebmann V., da Silva Nardi F., Wagner B., Horn P. A. (2014). HLA-G as a tolerogenic molecule in transplantation and pregnancy. *Journal of Immunology Research*.

[158] Lotti L. V., Mottola G., Torrisi M. R., Bonatti S. (1999). A different intracellular distribution of a single reporter protein is determined at steady state by KKXX or KDEL retrieval signals. *The Journal of Biological Chemistry*.

[159] Tang B. L., Ong Y. S., Huang B. (2001). A membrane protein enriched in endoplasmic reticulum exit sites interacts with COPII. *Journal of Biological Chemistry*.

[160] Scales S. J., Pepperkok R., Kreis T. E. (1997). Visualization of ER-to-golgi transport in living cells reveals a sequential mode of action for COPII and COPI. *Cell*.

[161] Larsen C. P., Elwood E. T., Alexander D. Z. (1996). Long-term acceptance of skin and cardiac allografts after blocking CD40 and CD28 pathways. *Nature*.

[162] Tan X., Zeng H., Jie Y., Zhang Y., Xu Q., Pan Z. (2014). CD154 blockade modulates the ratio of Treg to Th1 cells and prolongs the survival of allogeneic corneal grafts in mice. *Experimental and Therapeutic Medicine*.

[163] Gao W., Demirci G., Strom T. B., Li X. C. (2003). Stimulating PD-1-negative signals concurrent with blocking CD154 co-stimulation induces long-term islet allograft survival. *Transplantation*.

[164] Baas M. C., Kuhn C., Valette F. (2014). Combining autologous dendritic cell therapy with CD3 antibodies promotes regulatory T cells and permanent Islet allograft acceptance. *Journal of Immunology*.

[165] Goto R., You S., Zaitsu M., Chatenoud L., Wood K. J. (2013). Delayed anti-CD3 therapy results in depletion of alloreactive T cells and the dominance of foxp3^+^Cd4^+^ graft infiltrating cells. *American Journal of Transplantation*.

[166] Roelen D. L., van den Boogaardt D. E. M., van Miert P. P. M. C., Koekkoek K., Offringa R., Claas F. H. J. (2008). Differentially modulated dendritic cells induce regulatory T cells with different characteristics. *Transplant Immunology*.

[167] Hua J., Jin Y., Chen Y. (2014). The resolvin D1 analogue controls maturation of dendritic cells and suppresses alloimmunity in corneal transplantation. *Investigative Ophthalmology & Visual Science*.

